# Supplementary material for: ProteinShader: illustrative rendering of macromolecules
Source: BMC Struct Biol. 2009 Mar 30;9:19. doi: 10.1186/1472-6807-9-19 (PMC2672931; doi:10.1186/1472-6807-9-19)
Supplement: Additional file 1 — ProteinShader program without source code. This compressed file contains the complete ProteinShader program including associated libraries, but no source code. A README.txt file gives an overview of the ProteinShader distribution, and the index.html file in the help subdirectory has directions on getting started with the program as well as a set of tutorials. [file 1472-6807-9-19-S1.zip › ProteinShader-beta-0_9_4-binary/help/api/org/proteinshader/graphics/typography/GlyphSetFactoryFactory.html]

GlyphSetFactoryFactory (ProteinShader API)


|  |  |  |
| --- | --- | --- |
| **Overview** \| **Package** \| **Class** \| **Use** \| **Tree** \| **Deprecated** \| **Index** \| **Help** | |  |
| **PREV CLASS**   **NEXT CLASS** | **FRAMES**    **NO FRAMES**     **All Classes** |  |
| SUMMARY: NESTED \| FIELD \| CONSTR \| METHOD | DETAIL: FIELD \| CONSTR \| METHOD |  |


---


## org.proteinshader.graphics.typography Class GlyphSetFactoryFactory

```
java.lang.Object
  org.proteinshader.graphics.typography.GlyphSetFactoryFactory
```

---

``` public class GlyphSetFactoryFactory extends Object ```

Use Java reflection to load a concrete GlyphSetFactory based on
information held in a Properties object.

---

| **Field Summary** | |
| --- | --- |
| `static String` | `FONT_DIRECTORY`             Font files (glyph bitmaps) and their associated configuration file ('.conf' files) must be in the fonts directory, which has the relative pathname '/./../fonts/'. |


**Constructor Summary**

`GlyphSetFactoryFactory()`
  
          Constructs a TextLabelFactory.


| **Method Summary** | |
| --- | --- |
| `GlyphSetFactory` | `createGlyphSetFactory(String factoryName, String configName)`             Uses Java reflection to load the concrete GlyphSetFactory that is requested in the configuration file. |
| `Properties` | `readConfigFile(String configName)`             Reads the config file and stores its key-value pairs in a Properties object, which is a type of hash. |

**Methods inherited from class java.lang.Object**

`clone, equals, finalize, getClass, hashCode, notify, notifyAll, toString, wait, wait, wait`

**Field Detail**

### FONT\_DIRECTORY

```
public static final String FONT_DIRECTORY
```

:   Font files (glyph bitmaps) and their associated configuration
    file ('.conf' files) must be in the fonts directory, which
    has the relative pathname '/./../fonts/'.

    **See Also:**: Constant Field Values


**Constructor Detail**

### GlyphSetFactoryFactory

```
public GlyphSetFactoryFactory()
```

:   Constructs a TextLabelFactory.


**Method Detail**

### createGlyphSetFactory

```
public GlyphSetFactory createGlyphSetFactory(String factoryName,
                                             String configName)
                                      throws GlyphConfigException
```

:   Uses Java reflection to load the concrete GlyphSetFactory that
    is requested in the configuration file.

    :   **Parameters:**: `factoryName` - the name of the GlyphSetFactory to load.: `configName` - the name of the configuration file. **Returns:**: A concrete GlyphSetFactory for reading a '.glf' file. **Throws:**: `GlyphConfigException` - if an error occurs while trying to obtain the factory.

---


### readConfigFile

```
public Properties readConfigFile(String configName)
                          throws GlyphConfigException
```

:   Reads the config file and stores its key-value pairs in a
    Properties object, which is a type of hash.

    :   **Parameters:**: `configName` - the name of the configuration file to read. **Returns:**: A Properties object with the config file info. **Throws:**: `GlyphConfigException` - if an error occurs while trying to read the config file into the hash.


---


|  |  |  |
| --- | --- | --- |
| **Overview** \| **Package** \| **Class** \| **Use** \| **Tree** \| **Deprecated** \| **Index** \| **Help** | |  |
| **PREV CLASS**   **NEXT CLASS** | **FRAMES**    **NO FRAMES**     **All Classes** |  |
| SUMMARY: NESTED \| FIELD \| CONSTR \| METHOD | DETAIL: FIELD \| CONSTR \| METHOD |  |


---

# *Copyright © 2007-2008*
